# Forced labour risk is pervasive in the US land-based food supply

**DOI:** 10.1038/s43016-023-00794-x

**Published:** 2023-07-24

**Authors:** Nicole Tichenor Blackstone, Edgar Rodríguez-Huerta, Kyra Battaglia, Bethany Jackson, Erin Jackson, Catherine Benoit Norris, Jessica L. Decker Sparks

**Affiliations:** 1 Friedman School of Nutrition Science and Policy, Tufts University, Boston, MA USA; 2 Rights Lab, University of Nottingham, Nottingham, UK; 3 NewEarth B, York, ME USA

**Keywords:** Business and management, Agriculture

## Abstract

Social risk assessments and case studies of labour conditions in food production primarily focus on specific subpopulations, regions and commodities. To date, research has not systematically assessed labour conditions against international standards across diverse, complex food products. Here we combine data on production, trade, labour intensity and qualitative risk coding to quantitatively assess the risk of forced labour embedded in the US land-based food supply, building on our previous assessment of fruits and vegetables. We demonstrate that animal-based proteins, processed fruits and vegetables, and discretionary foods are major contributors to forced labour risk and that 62% of total forced labour risk stems from domestic production or processing. Our findings reveal the widespread risk of forced labour present in the US food supply and the necessity of collaborative action across all countries—high, middle and low income—to eliminate reliance on labour exploitation.

## Abstract (web summary)

The elimination of forced labour (Sustainable Development Goal 8.7) is a priority for the sustainability of food systems. Using data on production, trade, labour intensity and risk, this study estimates the risk of forced labour embedded in the US land-based food supply across product category, country of origin and supply chain stage.

## Main

Transformation of countries’ food systems is critical to achieving the United Nations Sustainable Development Goals (SDGs)^1^. Analysing the sustainability impacts or risks embedded in countries’ food consumption is an important lens for monitoring progress, particularly for policymakers. While some work has used a country-level lens to analyse aspects of the social performance of food systems^2,3^, no work to date has attempted to link social performance to particular food commodities at scale for countries. This level of resolution is critical to ensuring policy coherence as countries design targeted food systems interventions (for example, taxes) to achieve the SDGs while remaining within planetary boundaries.

Among SDGs relevant for the social sustainability of food systems, the elimination of forced labour (SDG 8.7) is a key priority. As defined by the International Labour Organization, “forced labor refers to situations in which persons are coerced to work through the use of violence or intimidation, or by more subtle means such as accumulated debt, retention of identity papers, or threats of denunciation to immigration authorities”^4^. The agriculture, fishing and forestry sector has one of the highest incidences of forced labour globally^5^. This sector relies on manual labour, often by migrant workers, who may be more vulnerable to deceptive and coercive practices^6^. While instances of forced labour are documented beyond the farm gate or dock, the incidence of forced labour in other food supply chain stages (for example, processing) is not quantified. The lack of data on forced labour (or any other labour-related risks) in recent analyses of sustainable food systems has the potential to create unintended consequences when translated to policy and practice.

The complexity of globalized supply chains and the illicit nature of forced labour present challenges for its detection and elimination^7^. However, with a rapidly evolving regulatory context that includes international trade sanctions and legislated human rights due diligence requirements, new supply chain approaches, data and indicators are needed to inform business^8^ and policy decision-making. Our previous work identified high risk of forced labour in the agricultural production of numerous fruit and vegetable commodities consumed in the United States^9^. The present paper builds on that social performance assessment, as a first step to understanding embedded labour-related risks across the diverse foods consumed in the United States. The objectives of this research were to (1) expand our forced labour risk scoring method to accommodate new data sources and the processing stages of food supply chains, accounting for global trade patterns; (2) estimate the risk of forced labour embedded in the diverse foods that compose a country’s food supply, using the United States as a case study; and (3) identify forced labour risk hotspots within and across food categories.

To compute forced labour risk, we first compiled origin data for the land-based US food supply (excluding seafood). Second, we qualitatively coded the forced labour risk in agricultural production and processing (where applicable) for each country–commodity combination using a three-tiered approach, with the most granular data available used in the final assessment (Table 1). Following the Social Hotspots Database (SHDB)^10^ approach, we applied conversion factors to translate qualitative risk levels into quantitative scores in the unit medium risk hours equivalent (mrh-eq). The risk of forced labour was calculated as a function of characterized risk and worker hours, and data quality was assessed using a pedigree matrix approach.

**Table 1:** Qualitative coding of forced labour risk levels

Risk level	Known occurrences (85% of level)			Government response (15% of level)
	Step 1: commodity–country	Step 2: sector–country	Step 3: country	
Very high	Commodity reportedly produced with forced labour; at least one account of forced labour	NA	NA	Tier 3 rank
High	Commodity is hand-harvested, and evidence of sector–country risk exists	Forced labour, debt bondage or labour trafficking occurs in the sector; at least one account or case of forced labour (explicitly noted)	>0.70% of people enslaved	Tier 2W rank
Medium	Concern/indicators of risk present and alleged conditions of forced labour	At least one report of forced labour, debt bondage or trafficking for labour in the sector; allegations and reports are noted	>0.30% of people enslaved	Tier 2 rank
Low	Conditions denoting risks of poor working conditions associated with vulnerability	Concern/indicators of risk present	>0.20% of people enslaved	Tier 1 rank
Very low	NA	NA	<0.19% of people enslaved	NA

The data sources for known occurrences (Step 1 (refs. ^16,17,67–71^), Step 2 (refs. ^16,72^) and Step 3 (ref. ^73^)) and government response^16^ were coded according to the schema below. More details on investigative journalism sources for Step 1 and Step 2 data can be found in the Supplementary Information. A weighted qualitative risk level for each observation was calculated as a function of known occurrences and government response. NA, not applicable.

## Results

**Fig. 1:**
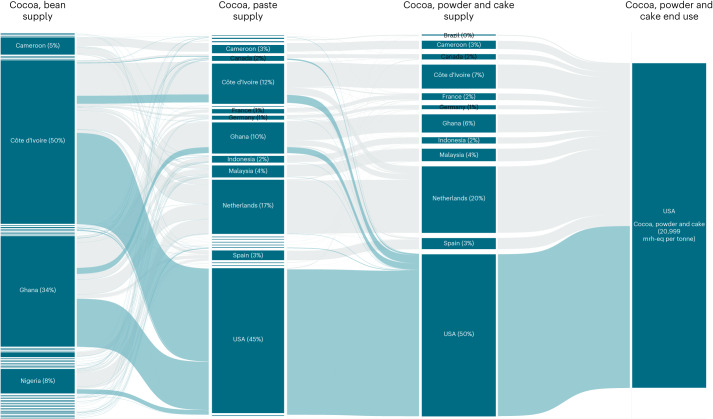
Distribution of forced labour risk by supply chain stage per tonne of cocoa powder and cake supplied to the United States. The flow of risk through supply chain stages and countries to supply cocoa, powder and cake to the United States. ‘Cocoa, bean supply’ is the agriculture stage; ‘cocoa, paste supply’ and ‘cocoa, powder and cake supply’ are the first and second stages of processing, respectively; and ‘cocoa, powder and cake end use’ refers to the consuming country (the United States). The percentages correspond to the percentage contribution to risk in each stage, as measured in the units mrh-eq per tonne. An interactive version of this figure showing all foods in the dataset is available at https://sites.tufts.edu/lasting/data/. Source data

Our final dataset included 212 food products and 1,312 product–country combinations (for example, orange juice from the United States). Of these product–country combinations, 41% were unprocessed food products (‘primary’, in the parlance of the Food and Agriculture Organization of the United Nations (FAO)), 48% included one stage of processing and the remainder had two (8%) or three (3%) stages of processing. We mapped processed products to estimated origin countries of the primary commodity using FAO’s Supply Utilization Accounts (SUA)^11^ and Detailed Trade Matrix^12^ (for example, orange juice from the United States is not assumed to be produced from only US oranges). Because of this complexity and the presence of products with multiple stages of processing, the total number of activity–country combinations—where ‘activity’ stands for a supply chain stage of a food product (for example, agriculture or first processing stage)—that were scored for risk in the final dataset was 2,661 (Supplementary Table 1). Figure 1 provides an example of the data structure^13^, illustrating how forced labour risk flows from multiple supply chain stages and countries of origin for the final food product consumed in the United States, cocoa powder/cake.

**Fig. 2:**
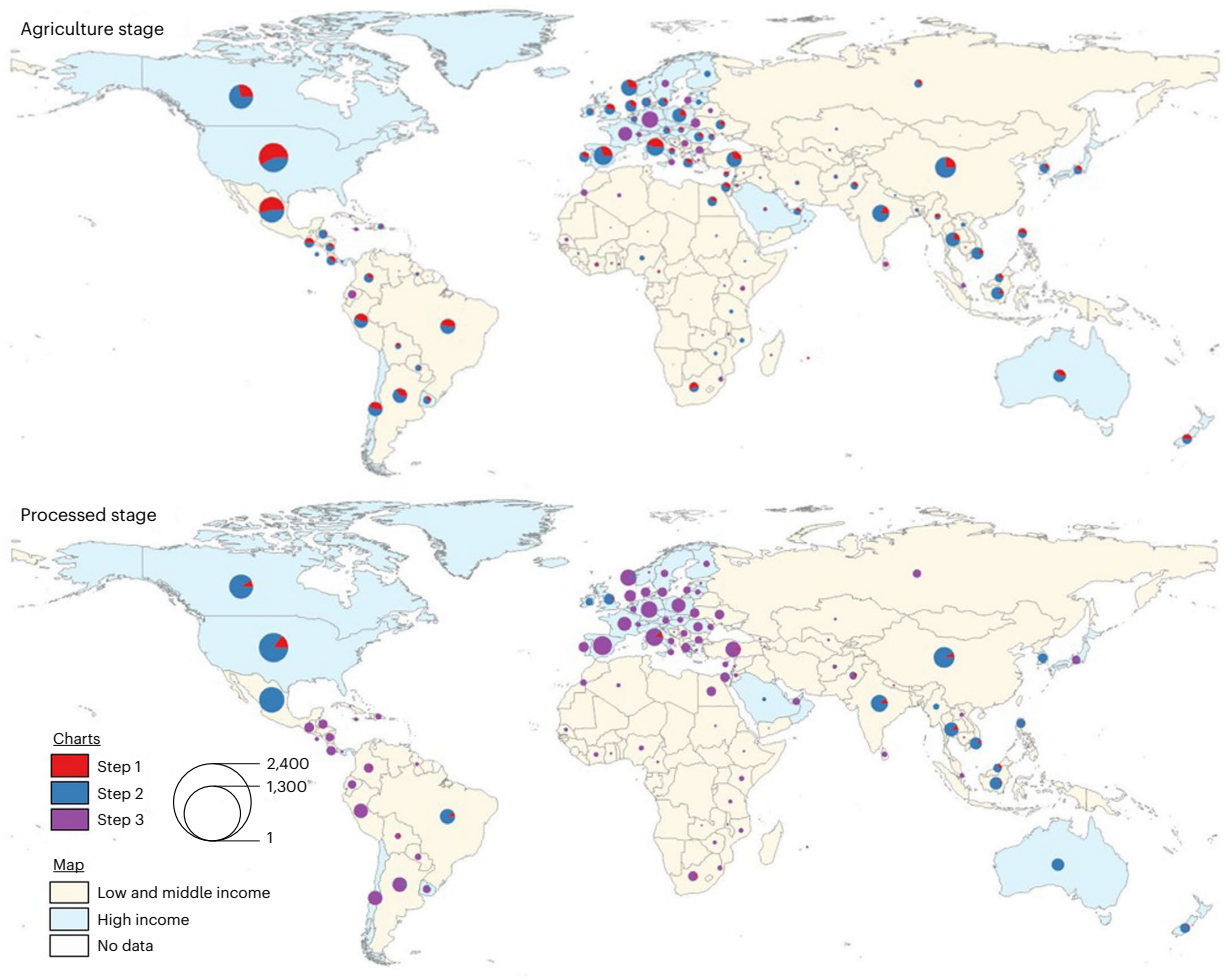
Resolution of forced labour risk data used for activity–country combinations in the final dataset, by stage of supply chain and country income. ‘Activity–country combination’ refers to an observation in the dataset that combines a supply chain stage or activity and a country of origin (for example, orange juice from the United States). Step 1 refers to commodity-specific risk, Step 2 refers to sector-specific risk and Step 3 refers to country-specific risk data. Credit: Basemap Source: ArcWorld Supplement, ESRI. Source data

Considering all activity–country combinations, 18% were scored for risk using commodity–country-specific data (that is, using Step 1 data; Table 1), 49% were scored using sector–country-specific data (Step 2 data) and 33% were scored using country-specific data (Step 3 data) (Supplementary Table 2). Activity–country combinations scored with commodity-specific data were equally distributed across high-income versus low- and middle-income countries (Fig. 2 and Supplementary Table 3).

**Extended Data Fig. 1:**
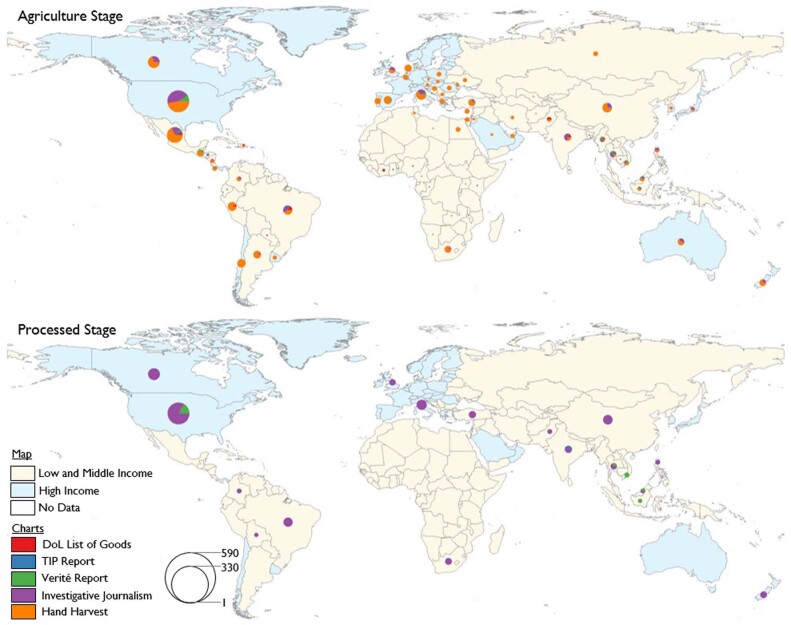
Distribution of Step 1 (commodity-country specific) data used for activity country combinations in the final dataset, by data source, stage of the supply chain, and country income. Activity-country combination refers to an observation in the dataset that combines a supply chain stage or activity and a country of origin (for example, orange juice from the US). Data sources listed are the U.S. Department of Labor’s List of Goods Produced by Child Labor or Forced labor (DoL List of Goods)^67^, U.S. Department of State’s Trafficking in Persons Report (TIP Report)^16^, Verité’s Strengthening Protections Against Trafficking in Persons in Federal and Corporate Supply Chains (Verité Report)^17^, Investigative journalism sources (see Supplementary Information), and hand harvest sources^68–71^. Credit: Basemap Source: ArcWorld Supplement, ESRI.

Focusing specifically on the agriculture stage of the supply chains, Step 1 risk data were available for 27% of combinations, spanning 81 countries (Fig. 2a and Supplementary Table 4). For these observations, 11% were from government and non-governmental organization (NGO) reports, 18% were from investigative journalism sources, and 71% were based on hand-harvest risk assessment (Extended Data Fig. 1 and Supplementary Table 5). The latter were estimated following methods described previously by Blackstone and colleagues^9^. The availability of commodity-specific data for the agriculture stage of products in our dataset ranged widely across countries, from 58% of combinations in the United States to no combinations in 69 countries, including several European nations. Sector-specific data for agriculture (Step 2) were available for 55% of combinations across 96 countries, with new data developed from investigative journalism sources accounting for 4% of sector-specific combinations (Supplementary Tables 4 and 5).

Much less Step 1 data were available for the processing stages of supply chains, at 4% of processing stage combinations (Fig. 2b and Supplementary Table 4). The most Step 1 data in processing was from the United States and Canada at 26% and 13% of combinations, respectively. Sector-specific data (Step 2) were available for 40% of the combinations for processed products (421 of processed products) across 25 countries.

### Hotspot analysis for the US food supply

**Fig. 3:**
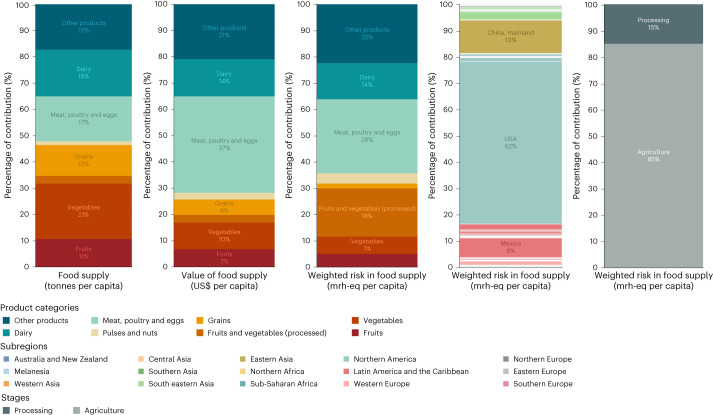
Quantity and value of the US land-based food supply versus embedded forced labour risk by product category, country of origin and supply chain stage. All data are presented on a per capita basis. The first two bars show the distribution of mass (in tonnes) and value (in US dollars) of the US land-based food supply by product category. The third bar shows the distribution of the risk of forced labour across product categories in the US land-based food supply, measured in mrh-eq and weighted by country of origin. The fourth bar shows the distribution of the risk of forced labour across countries of origin for the US land-based food supply, according to the last stage analysed in the supply chain for each food, measured in mrh-eq. The final bar shows the distribution of risk of forced labour by supply chain stage in the US land-based food supply, measured in mrh-eq and weighted by country of origin. Source data

We adapted a grouping schema by Kim et al.^14^ to analyse the distribution of forced labour risk across the land-based US food supply (Fig. 3 and Supplementary Table 7). The top three product categories that contributed to forced labour risk were meat, poultry and eggs (28%); other products (23%); and processed fruits and vegetables (18%). ‘Other products’ was a diverse category that included ‘discretionary foods’ such as sweeteners, beverages (coffee, beer and wine), chocolate and cocoa, among others. Processed fruits and vegetables included single-strength and concentrated juices as well as canned, frozen and otherwise preserved products. The other products and processed fruits and vegetables categories’ risk contributions were greater than their mass and economic value contributions, indicating disproportionately high risk. The meat, poultry and eggs category’s risk contribution was greater than its mass but not economic value contribution, indicating that the proportionality of risk was sensitive to the underlying food supply measure.

**Extended Data Fig. 2:**
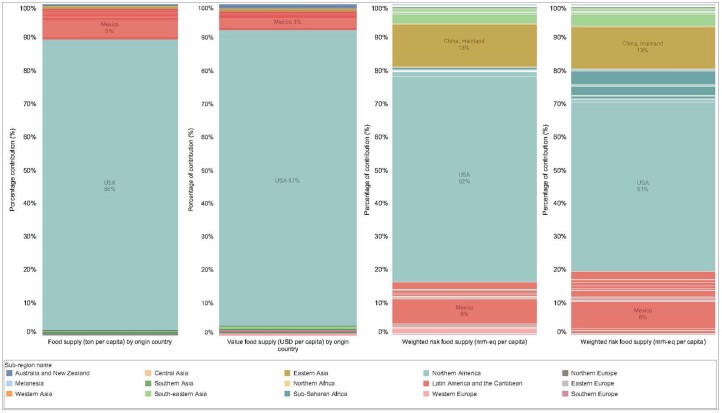
Quantity and value of the US land-based food supply versus embedded risk by country of origin. All data are presented on a per capita basis. The first two bars show the distribution of mass (in tons) and value (in US dollars) of the US-land based food supply by country of origin. The third bar shows the distribution of forced labor risk by country of origin in the US land-based food supply, measured in medium risk hours equivalent (mrh-eq). The fourth bar shows the distribution of forced labor risk by country of origin for the agriculture stage only, measured in mrh-eq. The country of origin for the first three bars corresponds to the last stage analysed for each food. The country of origin for the last bar corresponds to the agriculture stage only (first stage analysed).

Over half of the forced labour risk (62%) in the US land-based food supply was attributable to domestic production or processing (Fig. 3). While this is a substantial share, this is disproportionately low risk relative to the economic value and mass of domestic production in total (Extended Data Fig. 2). It is important to note that these risk, value and mass shares represent the last stage analysed in the supply chain; foods processed in the United States but grown in another country of origin would be classified as US. However, analysing risk by country of origin for agriculture only (that is, the first stage of the supply chain in our method) tells a similar story: 51% of the forced labour risk in agricultural production for the US supply was attributable to the United States (Extended Data Fig. 2).

**Extended Data Fig. 3:**
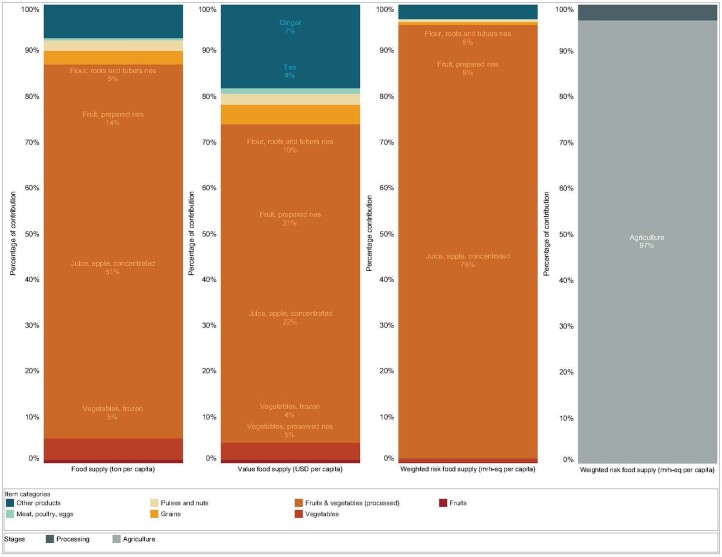
Quantity and value of imports from China in the US land-based food supply versus forced labor risk by product category and supply chain stage. All data are presented on a per capita basis. The first two bars show the distribution of mass (in tons) and value (in US dollars) of imports from China in the US land-based food by product category. The third bar shows the distribution of forced labor risk imported from China in the US land-based food supply, across product categories, measured in medium risk hours equivalent (mrh-eq). The final bar shows the distribution of forced labor risk imported from China in the US-land based food supply by supply chain stage, measured in mrh-eq.

**Extended Data Fig. 4:**
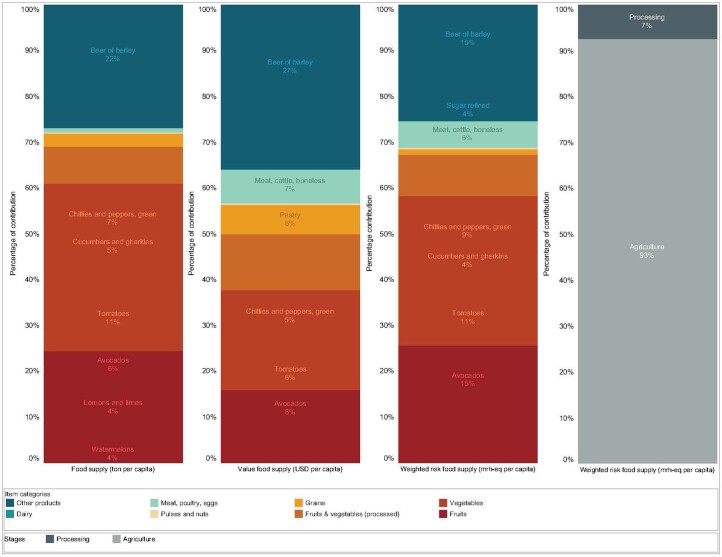
Quantity and value of imports from Mexico in the US land-based food supply versus forced labor risk by product category and supply chain stage. All data are presented on a per capita basis. The first two bars show the distribution of mass (in tons) and value (in US dollars) of imports from Mexico in the US land-based food by product category. The third bar shows the distribution of forced labor risk imported from Mexico in the US land-based food supply, across product categories, measured in medium risk hours equivalent (mrh-eq). The final bar shows the distribution of forced labor risk imported from Mexico in the US-land based food supply by supply chain stage, measured in mrh-eq.

The second and third highest contributing countries to forced labour risk in the US food supply were China and Mexico, at 13% and 8% of the total risk, respectively. The majority of China’s risk contribution was attributable to apple juice concentrate, accounting for 76% of forced labour risk sourced from China (Extended Data Fig. 3). China is the leading supplier of apple juice concentrate to the United States (60% of the supply), providing 766,830 tonnes per year in our timeframe (2015–2019). For Mexico, most of the imported risk was embedded in unprocessed fruits and vegetables, contributing 58% of the forced labour risk sourced from Mexico. These products primarily included avocados, tomatoes, and chillies and peppers (Extended Data Fig. 4).

**Extended Data Fig. 5:**
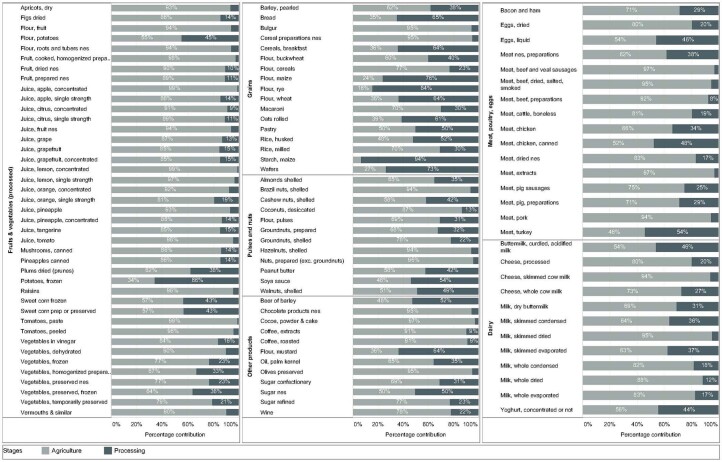
Distribution of forced labor risk product by supply chain stage. Forced labor risk is normalized to 100% for each product, showing the relative contribution of agriculture and processing stages to product-level risk. Risk is weighted by country of origin.

While the majority of risk embedded in the US food supply derives from agricultural production rather than food processing (85% versus 15%, respectively; Fig. 3), processing’s contribution to processed-product-level risk varied substantially, from 1% to 94%. Processing was a substantial contributor to per-unit risk for many products across food categories (Extended Data Fig. 5). For example, processing contributed 94% of the risk for maize starch, 66% for frozen potatoes, 52% for beer and 42% for shelled cashews. Proportionally high-risk contributions from the processing stage were due to higher coded risk from major supplying countries for processing relative to agriculture (for example, frozen potatoes), multiple processing stages (for example, maize starch) and/or higher labour intensity.

### Hotspot analysis for food categories

**Fig. 4:**
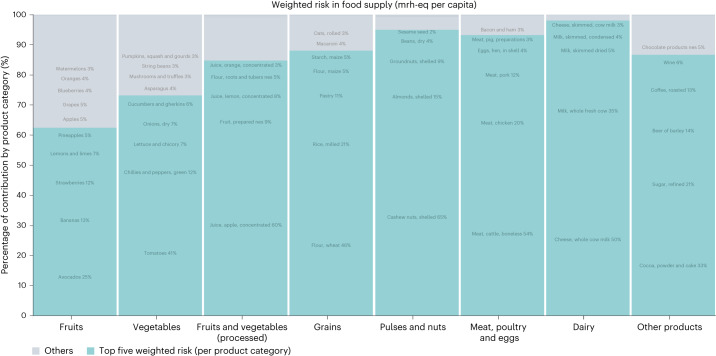
Contribution analysis of forced labour risk by product category, highlighting the top five products per category. The blue bar segments correspond to the top five products in each category according to their proportional contributions to forced labour risk per capita, measured in mrh-eq per capita and weighted by country of origin. The grey bar segments are all other food products in the category. Contributions less than 3% of the total in each bar are not labelled. NES, not elsewhere specified. Source data

**Extended Data Fig. 6:**
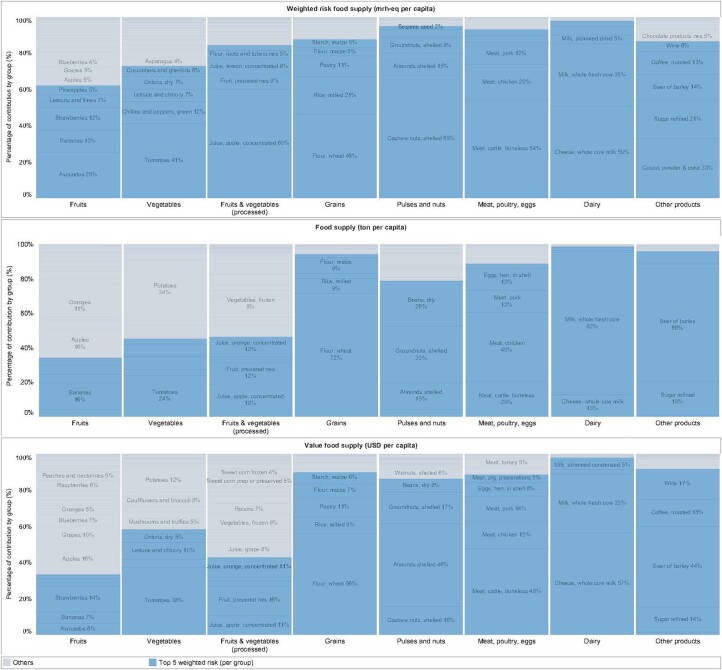
Food-level contributions of forced labor risk, mass, and value by product category. All values are per capita. Risk is weighted by country of origin and measured in medium risk hours equivalent (mrh-eq.).

**Extended Data Fig. 7:**
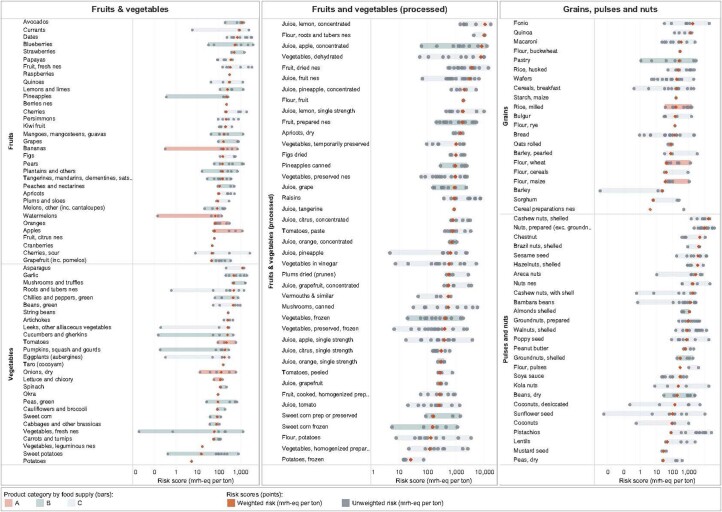
Food-level forced labor risk scores for produce, plant-based proteins, and grains. Forced labor risk scores are provided in the units medium risk hours equivalent (mrh-eq.) per ton for each food product for fruits, vegetables, processed fruits and vegetables, grains, and pulses and nuts. Points represent individual countries of origin (unweighted risk) or the average risk weighted by country of origin (weighted risk). The bar colors correspond to ranking by level of consumption, where (A) corresponds to products consumed at ≥5.2 kg per capita per year, (B) corresponds to products consumed at 0.8–5.1 kg per capita per year, (C) corresponds to products consumed at < 0.8 kg per capita per year. The ends of the bars correspond to the minimum and maximum risk scores for each product.

**Extended Data Fig. 8:**
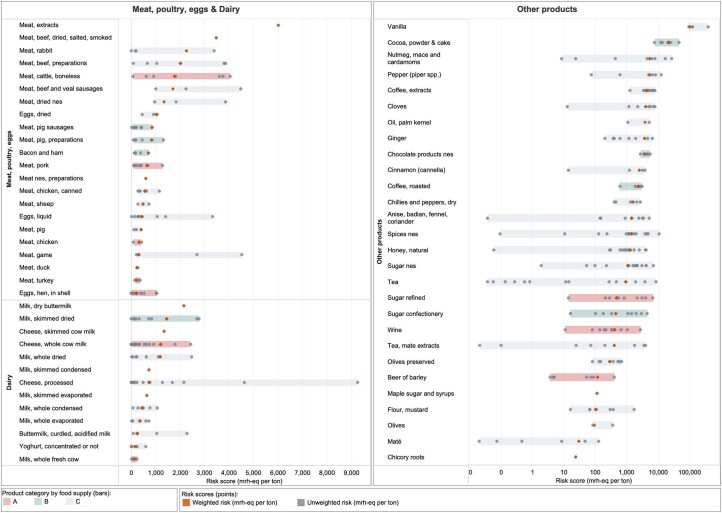
Food-level forced labor risk scores for animal-based foods and other products. Forced labor risk scores are provided in the units medium risk hours equivalent (mrh-eq.) per ton for each food product for meat, poultry, and eggs, dairy, and other products. Points represent individual countries of origin (unweighted risk) or the average risk weighted by country of origin (weighted risk). The bar colors correspond to ranking by level of consumption, where (A) corresponds to products consumed at ≥5.2 kg per capita per year, (B) corresponds to products consumed at 0.8–5.1 kg per capita per year, (C) corresponds to products consumed at <0.8 kg per capita per year. The ends of the bars correspond to the minimum and maximum risk scores for each product.

Within each of the analysed food categories, a small number of products contributed large shares of forced labour risk (Fig. 4). The contributions of the top five products by risk in each category ranged from 62% to 97% of food category risk, for fruits and dairy, respectively. Risk contribution relative to mass and value proportions of the food supply was variable; some products demonstrated disproportionate risk, and some did not (Extended Data Fig. 6). For clarity, we focus below on the top five products in each category that have disproportionately high risk relative to mass and economic value. We contextualize these hotspot results with risk results per unit of mass only (mrh-eq per tonne; Extended Data Figs. 7 and 8), as mass-based functional units (that is, denominators) are more commonly used in life cycle assessment and can be easier to interpret, especially in volatile price environments. Food categories are grouped by produce, plant-based proteins and grains, animal-based foods, and other products (the order is mirrored in Extended Data Figs. 7 and 8).

Among the top five fruits, those with disproportionately high risk included avocados, lemons and limes, and pineapples. These products are also high ranking in terms of risk on a mass basis, at weighted mean risk of 1,159 (avocados), 238 (lemons and limes) and 225 (pineapples) mrh-eq per tonne (Extended Data Fig. 7). Top vegetables with disproportionately high risk included tomatoes and chillies and peppers. Chillies and peppers was one of the highest-ranking vegetable products on a mass basis at a weighted mean of 434 mrh-eq per tonne; tomatoes were ranked sixth on a mass basis at 215 mrh-eq per tonne. For processed fruits and vegetables, apple juice concentrate was a leading contributor with disproportionately high risk. Apple juice concentrate also had the third highest risk per tonne for processed produce at a weighted mean of 7,779 mrh-eq. High risk is due to multiple factors, including the mass of apples required to produce concentrated juice (10 tonnes of apples per tonne juice concentrate^15^) and high reliance on imports from China.

For pulses and nuts, shelled cashews showed disproportionately high risk. Per tonne, shelled cashews had the highest risk among nuts and pulses, at 15,741 mrh-eq (Extended Data Fig. 7). The risk values and data quality ranged widely for this product, but Vietnam, which supplied 79% of shelled cashews for US consumption, was assessed as very high risk using commodity-specific data for agriculture and processing. Among all grain products, rice did not rank highly for risk on a per-tonne basis at 153 mrh-eq per tonne. However, among commonly consumed grains, it ranked third and showed substantial variability in risk depending on the country of origin.

Among the top contributors to food supply risk in the meat, poultry and eggs category, boneless beef demonstrates disproportionately high risk. On a per-tonne basis, boneless beef is high risk at 1,754 mrh-eq (Extended Data Fig. 8). About 90% of the supply for this product comes from the United States, which was coded as very high risk in the agriculture stage using commodity-specific data and high risk in the processing stage using sector data. For dairy, top products with disproportionately high risk included skimmed dried milk and skimmed cheese. Skimmed dried milk and cheese are high risk relative to other dairy products at 1,449 and 1,337 mrh-eq per tonne, respectively (Extended Data Fig. 8). Over 99% of the supply for these products comes from the United States, which was coded as very high risk using commodity-specific data for agriculture and as high risk in processing using sector data. Because the United States is the primary supplier for most dairy products, however, the high risk per unit mass relative to other dairy products was largely driven by product yields (approximately 10 tonnes of milk required per tonne of each product)^15^.

**Extended Data Fig. 9:**
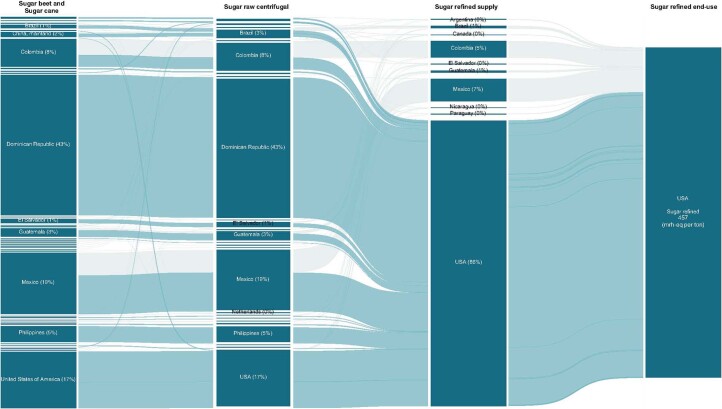
Distribution of forced labor risk by supply chain stage per ton of refined sugar supplied to the US. This figure illustrates how risk flows through supply chain stages and countries to refined sugar to the US. ‘Sugar beet and sugar cane supply’ is the agriculture stage, ‘sugar raw centrifugal’ and ‘sugar refined supply’, are the first and second stages of processing, respectively, and ‘sugar refined end-use’ refers to the consuming country (the US). Percentages provided correspond to the percentage contribution to risk in each stage, as measured in the units medium-risk hours equivalent (mrh-eq) per ton. An interactive version of this figure showing all foods in the dataset is available at https://sites.tufts.edu/lasting/data/.

Other top products with disproportionate risk were cocoa powder/cake and refined sugar. Cocoa powder/cake had the second highest forced labour risk per tonne in the dataset at 20,999 mrh-eq (Extended Data Fig. 8). Refined sugar was found to be the highest-risk sweetener in the dataset at 457 mrh-eq per tonne. Both cocoa powder/cake and refined sugar are complex products with two stages of processing and many origin countries (Fig. 1 and Extended Data Fig. 9). For cocoa, risk assessment for processing relied on sector- and country-level data, but risk at the agriculture/first stage included commodity-specific data for several key source countries, including Cote d’Ivoire and Ghana, which accounted for 84% of the agriculture stage risk for this product (Fig. 1). For refined sugar, commodity-specific risk data was available for processing for the United States (74% of the supply share for the end product), which was assessed as very high risk. Risk at the agriculture stage also included commodity-specific data for several key source countries, including the Dominican Republic, Mexico and the United States, which accounted for 79% of the agriculture stage risk for this product (Extended Data Fig. 9).

**Extended Data Fig. 10:**
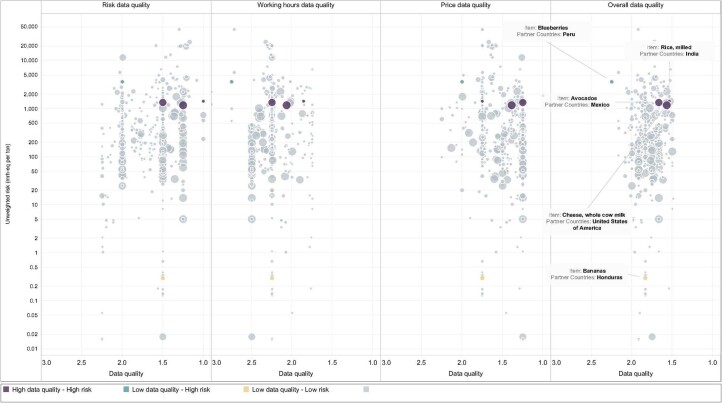
Data quality assessment results for risk, working hours, price, and overall. Each bubble corresponds to an end-product from a country of origin. Bubble size corresponds to the supply share for the country. Forced labor risk scores on the y-axis are in the unit medium risk hours-equivalent (mrh-eq.) per ton. Possible data quality scores range from 1 (high quality) to 5 (low quality). Axes stop at 3 because no observations in the dataset fell below a score of 3. Scoring matrices are provided in the Supplementary Information. Purple, blue, and yellow bubbles indicate example observations with high data quality and high risk scores, low data quality and high risk scores, and low data quality and low risk scores, respectively. An interactive version of this figure is available at https://sites.tufts.edu/lasting/data/.

Finally, the results of the data quality assessment indicated where commodity–country combinations fell along a continuum of quality, and thus how certain we may be of the results (Extended Data Fig. 10). Combinations with high data quality and high risk scores highlight opportunities to invest in primary data collection, document conditions and develop solutions in collaboration with workers. An example of high data quality and high risk is avocados from Mexico. Combinations with low data quality could be a target for more scan-level data collection, with the type of data improvement dependent on the sources of low data quality (that is, risk, price or working hours).

## Discussion

The forced labour risk assessment method developed and demonstrated here estimates risk at multiple supply chain stages (agriculture and multiple processing steps), including complex trade linkages, for diverse foods, while leveraging numerous sources for the triangulation of risk. Our findings for the US food supply revealed risk in more diverse food products than are typically identified in the literature, discussed by the media or NGOs, represented in government indices, or targeted by social responsibility initiatives. For example, while cocoa from Cote d’Ivoire and Ghana, cashews from Vietnam, and tomato products from Mexico exported to the United States have repeatedly been flagged as high risk for labour abuses^16,17^ (which we also identified), we found disproportionately high levels of risk in meat products produced in the United States. Because risk responses are determined by stakeholders’ perceptions of perceived risks^18^, methodological innovations that move beyond commodity case studies (which are important and necessary, but narrow in purview) may help eliminate geographic and commodity blind spots in supply chains. Our method can support more dynamic risk modelling^19^ and monitoring^20^ in supply chains without relying on intermittent social audits that have been critiqued for their inability to detect forced labour^21^.

At the level of the US food supply, we also found that a substantial fraction of forced labour risk was embedded within animal products, processed fruits and vegetables, and other products (that is, discretionary foods). These findings suggest areas of potential overlap (red meat, juices and refined sugars) and tension (tree nuts) with assessments of the environmental impacts and health outcomes associated with US food consumption^22–24^. Future research is needed to analyse these dynamics across all four pillars of sustainability (health, environmental, social and economic)^25^ for foods and diets, including trade-offs and synergies. As seafood was excluded from this analysis due to data limitations, future research should assess seafood as a potentially key point of tension for sustainability objectives in food systems. For example, increased seafood consumption, particularly of fish, is commonly recommended to promote human health^26^. Yet, like agriculture, fishing is known to have some of the highest risks of forced labour of any sector, owing to similar characteristics of the work (for example, high levels of manual labour) and the workforce (for example, frequent reliance on migrant workers)^27^.

Though it is often presupposed that most risk for high-income countries is embedded in importing practices and not domestic supply chains^16,28^, we found that more than half of the forced labour risk in US consumption can be attributed to domestic production or processing. The high fraction of domestic risk is both because the United States produces or processes a considerable fraction of what it consumes, on average, and due to systemic and long-standing risk in food-related labour. Contemporary forms of forced labour, debt bondage and labour exploitation are a continuation and evolution of a spectrum of labour abuses that began in US agriculture with chattel slavery and continued into forms of servitude post-Civil War into what they are today^29^. In high-income countries such as the United States, this has manifested as an overreliance on low-income migrant workers vulnerable to exploitation due to undocumented status or within the immigration programmes in which they are employed (for example, the H-2A visa programme for seasonal agricultural workers in the United States). These immigration programmes bind workers to a single employer, deny them access to the labour market^30^ and create multiple dependencies on employers that exacerbate vulnerability, such as transport between employer-supplied housing and fields and farms^31^. Furthermore, for countries where commodity or sector data were absent in our dataset, the country-level data that we relied on likely underestimated the risk in high-income countries. While international trade sanctions (including important bans such as US Customs and Border Protection’s Withhold Release Orders) are increasingly leveraged to reduce risks in globalized supply chains, our findings suggest that these should be just one tool in a larger strategy that harmonizes import controls with national and local regulation, monitoring, and enforcement to mitigate domestic risk. Perhaps a more robust method of action is the development of human rights due diligence frameworks, such as the European Union Corporate Sustainability Due Diligence Directive^32^, which seeks to address multiple aspects of the global value chain and require businesses to be liable for human rights and environmental abuses. The labour sector has been vocal in the need for redress around forced labour and has advocated for explicit language that draw links to forced labour within the legislation^33^.

Our findings underscore the widespread, systemic nature of forced labour risk in food systems. Eliminating forced labour and less severe forms of exploitation will require collaborative, worker-centred approaches, connecting macro quantitative risk assessments with worker communities on the ground to address power imbalances, legal loopholes, and regulatory enforcement challenges and limitations^34^. Numerous exploitative practices that do not reach the threshold of forced labour are normalized in agriculture, and understanding what decent work truly looks like from the worker community will also make it easier to detect and identify not just forced labour but all decent work deficits in quantitative assessments^31^. In the United States, the Fair Food Program for produce and Milk with Dignity for dairy have achieved marked improvements in working conditions for participating operations^35,36^. A Fair Fish pilot is underway in the United Kingdom, testing the model for seafood^37^. This model offers promise for achieving decent work for food system workers and is increasingly being critically examined to understand where and how it can be replicated and scaled with greater efficiency^38^.

Our approach represents a notable advance in social life cycle assessment (S-LCA) of food systems and labour risk assessment. Practice in S-LCA is typically bimodal, with scan-level assessments using sector- or country-level data^39,40^ or case studies^41–43^ in a particular context or for a particular company, although data for the latter are not often publicly available. While not a true cradle-to-gate S-LCA, our approach is an advance beyond our prior risk assessment method, which included only agriculture^9^. By integrating agriculture and processing, we probably capture the supply chain stages most likely to have high risks in food chains. At the same time, future research should explore novel data sources and risk associated with other stages of the supply chain, including animal feed production, transport^31^, retailing and waste management, to facilitate a high-resolution cradle-to-grave understanding of forced labour risks.

In the labour and human rights fields, there have increasingly been criticisms of built-in biases in risk assessment tools that ultimately rank countries against each other (for example, iterations of the Global Slavery Index)^28^ and/or use variable standards influenced by political objectives^44^. Specifically, concerns exist about biases and risks of absolving high-income countries of (1) accountability for their own domestic supply chains in-country and (2) responsibility for perpetuating the capitalistic structures that suppress wages and working conditions at the bottom of globalized supply chains^45^. Our approach prioritized assessing risk against standardized benchmarks (for example, relevant regulations and conventions), relying on country-level risk as a last resort, challenging traditional perceptions of where forced labour risk is embedded in food systems.

This expansion of our previously developed method^9^ includes investigative journalism reports, in addition to government, NGO and literature sources, as another data source to support risk triangulation. By incorporating investigative journalism, we were able to fill data gaps from previous analyses, including a more thorough assessment of risk in the processing stage and in US-based production and processing. Investigative journalism generally has lower evidence thresholds in terms of risk than official government reports. As a result, risk was captured in overlooked sectors and geographies, improving the dataset overall. Though our application of a rigorous coding approach addressed some of the quality limitations associated with using media reports as data sources, other inherent biases in reporting data remained. Forced labour is a hidden, illicit criminal activity; journalists need to know where to investigate and need to be able to access workers and sites. In particular, the former can be imbued by implicit biases, which manifest in terms of inequitable expectations for varied geographies and the persistence of racial tropes that influence interpretation and understanding of events. Media reports on exploitation and forced labour also under- or over-represent certain populations and demographics, under- or over-represent specific dimensions of forced labour, and often are framed around either foreignness or illegal immigration^46^.

We developed a data quality assessment framework to transparently address the use of risk data with varying levels of resolution and the use of multiple large databases to derive labour intensity for scaling risk. The results indicated that on average, data on working hours were of medium quality (Extended Data Fig. 10). Future research using a more rigorous and nuanced approach to quantifying working hours would improve the accuracy of the risk estimation. While working hours is a compelling variable to scale risk for S-LCA, there are limitations for labour risk assessment. Specifically, excessive overtime is an indicator of forced labour itself^47^. Excessive working hours and wage theft, which are intrinsically related, are typically the most commonly occurring dimensions of labour exploitation. Yet, they are frequently overlooked as indicators of risk since they may not reach the high evidentiary thresholds for forced labour on their own, particularly in countries where agricultural workers may be excluded from fundamental labour laws (for example, the United States). Improved data on working hours could therefore improve the utility of our approach for decision makers.

In conclusion, our method identified forced labour risk across diverse food products in the US food supply, from diverse countries of origin, including the United States. These findings are particularly salient considering the relatively sparse and unharmonious existing data on forced labour in food value chains and recent current events wherein US businesses proposed making additional trade data confidential^48^. Currently, industry drives and dampens the demand for data and metrics. While visibility should not be conflated with assurances, increasing demands from businesses for improved, comparable metrics and data on forced labour can help propel the shift from risk assessments towards accountability for harmed workers.

## Methods

The data were managed and analysed in Microsoft Excel (v.16.73), TableauPrep (v.2022.3.1) and TableauDesktop (v.2022.2.4). The overall calculation for forced labour risk per tonne of food product is described by equations (1) to (5):

1 $${\mathrm{labour}}\,{\mathrm{intensity}}{\left({\mathrm{h}}\,{\mathrm{t}^{-1}}\right)}_{i\,,j,k}={\mathrm{price}}{\left({\mathrm{US}}\$\,{\mathrm{t}^{-1}}\right)}_{i\,,j,k}\times {\mathrm{wh}}{\left({\mathrm{h}}\,{\mathrm{US}}\$^{-1}\right)}_{i\,,j,k}$$

2 $$\begin{array}{l}{\mathrm{risk}}\left({\mathrm{mrh}}{\mbox{-}}{\mathrm{eq}}\,{\mathrm{t}}^{-1}\right)_{i\,,j,k}\\={\mathrm{risk}}\,{\mathrm{characterization}}\,{\mathrm{factor}}_{i\,,j,k}\times {\mathrm{labour}}\,{\mathrm{intensity}}{\left({\mathrm{h}}\,{\mathrm{t}}^{-1}\right)}_{i\,,j,k}\end{array}$$

3 $${\mathrm{unweighted}}\,{\mathrm{risk}}\left({\mathrm{mrh}}{\mbox{-}}{\mathrm{eq}}\,{\mathrm{t}}^{-1}\right)_{i\,,j}={\sum }_{k=1}^{n}{\mathrm{risk}}_{i\,,j,k}\times {\mathrm{e{R}}}_{j,k}\times {\mathrm{share}}_{i\,,j,k}$$

4 $${\mathrm{weighted}}\,{\mathrm{risk}}\left({\mathrm{mrh}}{\mbox{-}}{\mathrm{eq}}\,{\mathrm{t}}^{-1}\right)_{j,l}={\sum }_{i=1}^{n}{\mathrm{unweighted}}\,{\mathrm{risk}}_{i\,,j}\times {\mathrm{share}}_{i\,,j,l}$$

where each final food product is denoted *j*, each supply chain stage is denoted *k*, each country of origin is denoted *i* and the consuming country is denoted *l*. Overall, each food product *j* consumed in country *l* (here, the United States) consists of the integration of one or more supply chain stages *k* from origin countries *i* to meet the total consumption.

Risk per unit output (equation (2)), defined as each possible combination among origin country *i*, food product *j* and supply chain stage *k*, is estimated by multiplying the risk characterization factor (in the unit mrh-eq) for that combination by its respective labour intensity (equation (1)). The unweighted risk for each food product *j* from origin country *i* (equation (3)) is then calculated by adding the risk for each supply chain stage *k* multiplied by its corresponding extraction rate (eR), and the supply share from each country of origin at supply chain stage *k*, where supply chain stage *k* ranges from 1 to *n* (equation (4)), defined by food product *j*’s respective commodity tree (see below). Finally, the mean weighted risk (equation (5)) is equal to the sum of the unweighted risk multiplied by the proportion or share of consumption from each country *i* respecting each food product *j* sourced by country *l*, where *i* ranges from 1 to *n*, defined by the number of origin countries providing >1% of the supply for that specific product.

The weighted risk embedded in per capita food consumption is then calculated by multiplying the weighted mean risk of that product by the per capita food supply (equation (5)):

5 $$\begin{array}{l}{\mathrm{risk}}\,{\mathrm{per}}\,{\mathrm{capita}}\left({\mathrm{mrh}}{\mbox{-}}{\mathrm{eq}}\,{\mathrm{per}}\,{\mathrm{capita}}\right)_{j,l}\\={\mathrm{weighted}}\,{\mathrm{risk}}\left({\mathrm{mrh}}{\mbox{-}}{\mathrm{eq}}\,{\mathrm{t}}^{-1}\right)_{j,l}\times {\mathrm{food}}\,{\mathrm{supply}}\left({\mathrm{t}}\,{\mathrm{per}}\,{\mathrm{capita}}\right)j,l\end{array}$$

where *l* refers to the consuming country (that is, the United States, in this application) and *j* refers to the consumed food product.

### US food supply and origins

We selected FAOSTAT’s SUA^11^ as the main data source for estimating the US supply, which includes imported commodities as well as domestically produced ones. Because the SUA database aggregates imports (at the partner country level), we incorporated import values from the FAO Detailed Trade Matrix^12^. We averaged values and quantities for 2015–2019 to smooth interannual variability.

In total, there are 806 potential SUA items, which results in 351 commodities with supply data in the United States. However, not all are used as food. In addition, in the data cleaning process, several commodities were identified that do not have sufficient volume or that have relevant missing data that forced us to eliminate them from the final set of commodities (that is, by-products or complex products such as ‘infant food’). Once we obtained a subset of supply items, we filtered again considering only the commodities that are utilized as food. We thus present the weighted risk for food use items in the SUA, while the risk for the processed use is embedded in the supply chain stages *k*. In the end, we present 239 unique products, of which 211 are end products. The remaining 28 products are products that are not consumed as food directly but are used as raw material (such as wheat, cocoa beans and sugar cane) for processed food products. We use the term ‘food supply’ as shorthand for ‘food utilization’, a proxy measure for food consumption within a country.

SUA uses commodity trees to associate primary items with processed products. A commodity tree includes the extraction rates (the rates of conversion of the processed product to the primary product), the primary-to-child-item relationship (which primary product is needed to produce a processed commodity) and the parent-to-child-item relationship (the level immediately prior to the end product). If the primary product is different from the parent, this means that the processed product has more than one upstream level, since more than one processed step is required to produce it (for example, wheat flour and wheat pastry). In the database used for the United States, we identified a maximum of three levels of upstream. Although FAO mentions that commodity trees and extract rates are variable across countries and times, we used general commodity trees and extract rates^49^ (Supplementary Table 8). Commodity trees do not connect primary animal products with feed production upstream; feeds were not included in this risk analysis.

### Prices

To estimate the labour intensity, unit values (producer prices) are needed. However, there are substantial gaps in data availability for producer prices. FAO producer prices^50^ are available only for primary commodities (only some commodity–country pairs). The FAO trade database also includes export and import prices, which have better coverage within our database but include additional markups (for example, for transport) beyond the producer price (that is, import prices are based on cost + insurance + freight prices, and export prices are based on freight-on-board price). To estimate price data for each commodity–country combination, a data hierarchy was established, and Global Trade Analysis Project data at the country–sector level were used to estimate correction factors for the most accurate price estimates possible.

### Labour intensity

Labour intensity per tonne (worker hours per tonne) is estimated for each food product *j*, supply chain stage *k* and country *i* combination, as a function of its respective price (US$ per tonne) and working hours per unit value (working hours per US$). We used data on working hours (worker hours per US$1 of country-specific sector output) from the SHDB^10^, previously described by Blackstone and colleagues^9^. The sectors in the SHDB come from the Global Trade Analysis Project database.

### Forced labour data sources

Forced labour risk was constructed through a multi-step process wherein risk was qualitatively coded using data on known occurrences and government responses (Table 1). Known occurrence data required the use of numerous sources to cover all country–commodity combinations and was sorted by resolution in three steps. Step 1 was commodity–country-specific risk, Step 2 was sector–country-specific risk and Step 3 was country-specific risk. Risk from the highest-resolution step of data available was used for the final quantitative score. Data on country-level government response were taken from the Trafficking in Persons Report^16^. A final qualitative code was developed for each observation that accounted for known occurrences (85%) and government response (15%). Further details are provided below.

The sources for Steps 1–3 included US government reports, NGO reports and several sources on harvest methods, as described in our previous work^9^. We also constructed a dataset using investigative journalism sources to fill numerous risk data gaps. We used Nexis Uni^51^ to conduct a search using a base set of labour-related terms and commodity or processing terms (Supplementary Information). The cut-off range used was 2016–2019, inclusive, to account for the enactment of the SDGs and to prevent the inclusion of COVID-19-related articles. Within the results, we excluded state-run media, advertisements and opinion pieces. In total, one reviewer screened 38,207 articles for relevance regarding labour conditions, forced labour or human trafficking for labour. When duplicates were identified, articles with the most reputable outlets with the largest circulation were retained for coding. The final sample of articles (*n* = 709) was double-coded on the basis of a codebook developed for assessing the risk of forced labour in fruits and vegetables^9^ and adapted and expanded to fit the needs of this project. Prison labour was also coded as very high risk due to the often forced nature of the work being tied to punishment and used as a coercive tactic to reduce sentence length. Prison labour is often not explicitly included in the definition of forced labour, yet we consider its risks important to include in our analysis because many goods produced end up in the food supply for profit over rehabilitation, particularly in the United States, where prison labour is rooted in the 13th Amendment^52^. For a more detailed description of the process, see the Supplementary Information.

### Qualitative coding of forced labour risk levels

Paired researchers coded each data source independently using the aforementioned codebook. For known occurrences, risk coding for all steps of data followed our previously developed coding schema, outlined in Blackstone and colleagues^9^ and in Table 1. New codes and methods were created for investigative journalism data sources. Investigative journalism articles were read in their entirety, and the corresponding countries and commodities (Step 1) or sectors (Step 2) were identified. For sources that included commodity–country-specific data (Step 1), commodity–country combinations were coded as very high risk, medium risk, low risk or not applicable. For sources that included sector–country-specific data (Step 2), sector–country combinations were coded as high risk, medium risk, low risk or not applicable. If a commodity was not included in any of the above reports, risk was not assessed, as exclusion did not equate to no risk. A percentage of interrater agreement was calculated (Supplementary Information) to assess the consistency of the two coders’ deductive application of the ordinal risk rating scale^9^, since absolute agreement was ultimately necessary for the code to be used in risk assessment. While there is no universally accepted threshold for high or low percentage agreement, agreement for each step and data source exceeded the minimum percentages of 75–80% frequently referenced^53^; however, agreement may have been overestimated by not accounting for chance^54^.

A single known-occurrences risk code was identified for each country–commodity combination by taking the highest risk code if multiple codes were present. Where this was not the case, a mini-Delphi approach^55–58^ (a method commonly used in the health sciences) was undertaken with five experts from the research team who read through the initial codes and justifications before coming to a consensus aligned with the codebook on the overall risk code. This was undertaken for 39 country–commodity combinations (Step 1) and 10 sector–country combinations (Step 2).

Government response data were taken from the US Trafficking in Persons Report^16^ and coded as very high, high, medium or low risk, or not applicable, following our previous methods^9^. Finally, a weighted average qualitative risk level was constructed following SHDB^10^ methods and our earlier approach^9^, where known-occurrences data are weighted at 85% and governance is weighted at 15% of the final level (Supplementary Information). When either known occurrences or government response data were unavailable, risk was assessed using the highest-resolution data available. Overall, we took a conservative approach to risk assessment, structuring the coding schema to reflect uncertainty. For example, for known occurrences, a ‘very high’ risk code was only used for commodity–country-specific data and a ‘very low’ code was only used for country-specific data.

### Quantitative scoring of forced labour risk

We used a reference scale S-LCA approach, where the aim is to assess social risk or performance^59^. After computing the weighted average qualitative risk level, we applied characterization factors to convert to the unit mrh-eq for each observation. Used by the two major S-LCA databases (that is, SHDB^10^ and PSILCA^60^), this unit enables straightforward, scalable comparisons across products and the identification of hotspots within a sourcing portfolio or supply chain. The SHDB^10^, produced by NewEarth B, was the first available S-LCA database and has pioneered many of the methods^61^ and best practices now enshrined in the S-LCA Guidelines^62^. As such, we adapted the SHDB social impact assessment (that is, characterization) method^10^, using the following conversion factors: very high risk = 10 mrh-eq, high risk = 5 mrh-eq, medium risk = 1 mrh-eq, low risk = 0.01 mrh-eq, very low risk = 0.001 mrh-eq^9^. Unlike characterization factors in environmental LCA, which reflect a causal pathway between flow (for example, methane emissions) and outcome (for example, global warming potential), the connection between working hours and forced labour is not causal. However, the duration of working time needed is a compelling variable to use to scale and compare social risks.

### Data validation and data quality assessment

For price, working hours and labour intensity data, we preprocessed the data, identifying values outside of the 5th and 95th percentiles and normalizing with a Winsorization approach^63^. Outliers below and above these thresholds were substituted with the 5th and 95th percentile values, respectively.

We developed a data quality assessment framework specific to our application (Supplementary Information) by adapting the S-LCA pedigree matrix^64^, recommended by the 2020 S-LCA Guidelines^62^, and a recent version of the pedigree matrix used in environmental LCA^65,66^. We constructed pedigree matrices using the same four indicators (reliability, temporal, geographical and technical) for each major data component for the analysis: risk coding, working hours and prices. The indicators were assessed at five levels, from 1 (meaning very good performance) to 5 (meaning very poor performance). The final data quality score for each observation was calculated by averaging the scores across the four indicators for each data source and then averaging the scores across each data source.

### Reporting summary

Further information on research design is available in the Nature Portfolio Reporting Summary linked to this article.

## Supplementary information

Supplementary Information Supplementary description of the methods referenced in the text.

Reporting Summary

Supplementary Tables 1–10 Supplementary tables referenced in the manuscript.

## Data Availability

The detailed results and background data files are available for download at https://dataverse.harvard.edu/dataverse/lasting, and interactive visualizations of select results are available at https://sites.tufts.edu/lasting/data/. The supply and origin data that support the findings of this study are available from FAO (http://www.fao.org/faostat/en/#data and https://github.com/SWS-Methodology/faoswsAupus). The price data that support the findings of this study are available from FAO (http://www.fao.org/faostat/en/#data) and the Global Trade Analysis Project (https://www.gtap.agecon.purdue.edu/databases/v8/). The forced labour and governance data that support the findings of this study are available from the US Department of Labor, Bureau of International Labor Affairs (https://www.dol.gov/agencies/ilab); the US Department of State, Bureau of Democracy, Human Rights, and Labor and Office to Monitor and Combat Trafficking in Persons (https://www.state.gov/); Verité (https://www.verite.org/); and the Walk Free Foundation (https://www.globalslaveryindex.org/about/the-index/). Source data are provided with this paper.

## Source data

Source Data Fig. 1 Tabular data supporting Fig. 1.
Source Data Fig. 2 Tabular data supporting Fig. 2.
Source Data Fig. 3 Tabular data supporting Fig. 3.
Source Data Fig. 4 Tabular data supporting Fig. 4.

## Code availability

Data processing and analysis were performed using Tableau Prep (v.2022.3.1) and Tableau Desktop (v.2022.2.4). TFL (Tableau Prep) files are available from the corresponding author upon reasonable request.
