# Supplementary material for: Forced labour risk is pervasive in the US land-based food supply
Source: Nat Food. 2023 Jul 24;4(7):596–606. doi: 10.1038/s43016-023-00794-x (PMC10365991; doi:10.1038/s43016-023-00794-x)
Supplement: Supplementary file 1 — Supplementary description of the methods referenced in the text. [file 43016_2023_794_MOESM1_ESM.pdf]

nature food

Article https://doi.org/10.1038/s43016-023-00794-x

Forced labour risk is pervasive in the US land-based food supply

In the format provided by the authors and unedited

Contents

Investigative Journalism Methods 1
Investigate Journalism Search Terms Used 2
Interrater Reliability 6
Weighted Average Qualitative Risk Level Calculation 6
Data Quality Assessment Frameworks 7
Data quality assessment framework for risk coding 7
Data quality assessment framework for working hours 8
Data quality assessment framework for price data 8
Combined data quality assessment framework for risk coding, working hours and price 8

Investigative Journalism Methods

We conducted a search on NexisUni with a uniform set of base search terms that encompass labor conditions and forced labor as well as specific commodities and processing terms (see below). Specific commodities had their own exclusion words (ex: for "banana", we excluded "banana republic"); there were no generalized exclusion criteria for the initial search. In total, 117 commodities and 17 processing terms were searched to yield a total initial n of 86,116 news articles. The initial cut-off date range for the search was 2011-2021.

We implemented new cut off dates of 2016-2019, inclusive to account for the enactment of the Sustainable Development Goals at the UN level on January 1, 2016 and to ensure that primarily pre-COVID articles were included, where the first COVID case was reported at the end of 2019 (December 31, 2019).[1] This decreased the n to 38,207 articles. We excluded all state-run media to prevent misrepresentation from propaganda on our results by utilizing a list of state-run media published by Wikipedia to determine which sources were inappropriate to reference. We also excluded both opinion pieces and advertisements.

One reviewer performed a first-stage screening of the headlines and articles to determine relevance of poor labor conditions, instances of human trafficking for labor, and reported/alleged/risk of forced labor in a commodity-country or sector-country combination. Articles and their duplicates considered irrelevant were deleted. In remaining group of relevant articles, the duplicates were removed, and we recorded what commodity or sector was mentioned

and whether it was at the farm gate or at the processing stage. In instances where duplicates came from different publications, we retained the publication with the largest circulation that was most reputable. In total, 38,207 articles were reviewed, of which 709 articles were retained for coding (Supplementary Table 9 and 10). There are instances where multiple articles discuss the same commodity-country or sector-country combination, though with different language to describe the working conditions or indicators of forced labor. We kept all duplicates of those articles to ensure a robust collection for coding that accounts for potential instances of codes varying amongst a commodity- or sector- country combination.

These articles were coded by two coders based on a codebook developed for assessing the risk of forced labor in fruits and vegetables[2] and adapted and expanded to fit the needs of this project. Disagreements in codes were discussed until a consensus was reached. Final country-commodity and sector-commodity combinations were reviewed and consolidated to remove duplicates prior to determining the final code. To get the final codes for step 1 and step 2, the highest-level code was taken if there were multiple of that code. If there were not multiple, a mini-Delphi approach was utilized to determine the final code for that combination. There was a final review for duplicates after final codes were determined whereby the highest code for each commodity-country and sector-country combination was kept. Note: seafood species were included in the search and coding process but excluded from the analysis due to lack of comprehensive trade and origin data from FAO.

Investigate Journalism Search Terms Used

**List of Fresh and Processed Commodities and Terms used in Search**	
**Database Searched**:<br>Nexis Uni	**Base Search Terms**:<br>("forced labor" OR "forced labour" OR "debt bondage" OR (labour w/2 abuse!) OR (labor w/2 abuse!) OR (modern w/2 slave!) OR (labor w/2 exploit!) OR (labour w/2 exploit!) OR "migrant worker" OR (human w/2 traffick!))
**Dates**: 2011-2021	
**Commodity**	**Commodity Search Terms** (AND -)
**Fruit, Vegetables, Livestock:**	
Almonds	(“almond” OR “almonds”)
Apples	(“apple” OR “apples”)
Apricots	(“apricot” OR “apricots”)
	Ex: “apricot seed”, “apricot flowers”, “apricot lane”, “apricot lane boutique”
artichokes	(“artichoke” OR “artichokes”)
Avocado	(“avocado” OR “avocados”)
Banana	(“banana” OR “bananas”)
	Ex: “banana republic”
Barley	(“barley”)
Beans	(“bean or beans”)
	Ex:<br>"cocoa bean/(s)", "coffee bean/(s)", "L. L. Bean", "cacao bean(s)"
Beef	(“beef” OR “veal” OR “bull”)

	Ex: "Bull market", "Tom Bull"
blackberries	(“blackberry” OR “blackberries”)
blueberries	(“blueberry” OR “blueberries”)
Butter	(“butter”)
	Ex: "cocoa butter", "cocao butter", "peanut butter", "shea butter"
cabbages	(“cabbage” OR “cabbages”)
canola_oil	(“canola” OR “canola oil”)
	Ex: “palm oil”
carrots_turnips	(“turnip” OR “turnips”)
Cassava	("cassava" OR "manioc" OR "yuca")
cauliflower_broccoli	("cauliflower" OR "cauliflowers" OR "broccoli" OR "broccolis")
cereals_other	("quinoa" OR "popcorn" OR "pop corn" OR "buckwheat" OR "fonio" OR "triticale" OR "canary seed" OR "mixed grain" OR "flour cereal" OR "bran cereal")
cherries	(“cherry” OR “cherries”)
coconut	(“coconut” OR “coconuts”)
	Ex: "palm oil", "monkey", "monkeys"
coconut_oil	(“coconut oil”)
corn	(“corn” OR “maize”)
	Ex: “palm oil”
corn_oil	(“corn oil”)
cottonseed_oil	(“cottonseed oil”)
cranberries	(“cranberry” OR “cranberries”)
cream	(“cream”)
crustaceans_other	("crustacean" OR "crustaceans")
cucumbers	(“cucumber” OR “cucumbers”)
eggplant	(“eggplant” OR “eggplants”)
eggs	(“egg” OR “eggs”)
	Ex: “easter egg”, “easter eggs”
figs	(“fig” OR “figs”)
freshwater_fish	(“freshwater fish”)
garlic	(“garlic” OR “garlics” OR “scapes”)
grapefruit	(“grapefruit” OR “grapefruits”)
grapes	(“grape” OR “grapes”)
green_beans	("green bean" OR "green beans" OR "snap bean" OR "snap beans” OR “lima bean” OR “lima beans”)
green_peas	("green pea" OR "green peas" OR "snap pea" OR "snap peas")
groundnut_oil	("peanut oil" OR "groundnut oil" OR "arachis oil")
groundnuts	("peanut" OR "peanuts" OR "monkey nuts" OR "monkey nut" OR "goober" OR "goobers" OR "pindar" OR "pindars" OR “groundnuts” OR “groundnut”)
hazelnuts	(“hazelnut” OR “hazelnuts”)
honey	(“honey”)
kiwi	(“kiwi” OR “kiwis”)

lettuce	(“lettuce”)
mangoes	(“mango” OR “mangoes”)
marine_fish	(“marine fish”)
milk	(“milk” OR “dairy” OR “dairies”)
millet	(“millet”)
molluscs	("mollusc" OR "molluscs")
mutton	("mutton" OR "lamb" OR "lambs")
nuts_other	
chestnut	(“chestnut” OR “chestnuts”)
brazil nut	(“brazil nut” OR “brazil nutes”)
cashew	(“cashew” OR “cashews” OR “cashew nut” OR “cashew nuts)
oats	(“oat” OR “oats” OR “oatmeal”)
offals	("offals" OR "variety meats" OR "organ meats" OR "pluck")
oilcrops_other	("rape seed" OR "mustard seed" OR "rapeseed")
	Ex: "palm oil", "canola"
oils_other	N/A
okra	(“okra”)
olive_oil	(“olive oil”)
olives	(“olive” OR “olives”)
	Ex: “olive garden”, “olive oil”, “Mt. Olive”
onions	(“onion” OR “onions”)
oranges	(“orange” OR “oranges”)
palm_oil	(“palm oil”)
palmkernel_oil	(“palm kernel oil”)
papayas	(“papaya” OR “papayas”)
pelagic_fish	("pelagic fish" OR "pelagic fishes")
pineapples	(“pineapple” OR “pineapples”)
pistachios	("pistachio" OR "pistachios")
plantains	("plantain" OR "plantains”)
plums	(“plum” OR “plums”)
pork	("pork" OR "bacon" OR "hog" OR "ham" OR "panchetta" OR "pork belly")
potatoes	("potato" OR "potatoes")
poultry	(“chicken” OR “chickens” OR “poultry” OR “turkey” OR “turkeys” OR “duck” OR “ducks”)
pulses	("lentil" OR "lentils" OR "vetches" OR "lupins" OR "peas" OR "pea")
pumpkin_squash	(“pumpkin” OR “pumpkins” OR “squash” OR “squashes”)
raspberries	(“raspberry” OR “raspberries”)
rice	(“rice”)
roots_other	("yautia" OR "cocoyam" OR "taro")
rye	(“rye”)
sesame	(“sesame” OR “sesame seed” OR “sesame seeds”)
sesameseed_oil	(“sesame seed oil” OR “sesame oil”)

sorghum	(“sorghum”)
soybean_oil	(“soybean oil”)
soybeans	(“soybean” OR “soybeans”)
spinach	(“spinach”)
sugar	(“sugar”)
sugar_beets	("sugar beet" OR "sugar beets")
sugar_cane	("sugar cane" OR "sugarcane" OR "sugar canes" OR "sugarcanes")
sunflower_seeds	(“sunflower seed” OR “sunflower seeds”)
sunflowerseed_oil	(“sunflower seed oil”)
sweet_corn	(“sweet corn”)
sweet_potatoes	(“sweet potato” OR “sweet potatoes”)
sweeteners_other	("fructose" OR "maltose" OR "maple sugar" OR "molasses" OR "other fructose" OR "glucose" OR "dextrose" OR "lactose" OR "isoglucose")
tomatoes	(“tomato” OR “tomatoes”)
vegetables_other	
Asparagus	(“asparagus”)
Carrot	(“carrot” OR “carrots”)
Pepper	(“pepper” OR “peppers”)
Mushroom	(“mushroom” OR “mushrooms”)
walnuts	(“walnut” OR “walnuts”)
	Ex: "walnut care at home", "walnut grove secondary", "walnut valley baptist church", "walnut St."
wheat	(“wheat”)
**Fish:**	
Shrimp	(“shrimp”)
Crab	(“crab” OR “crabs”)
	Ex: "fields like crabs"
Tuna	(“tuna” OR “canned tuna” OR “white albacore tuna” OR “skipjack tuna” OR “tongol” OR “big-eye” OR “yellow fin”)
Cod	(“cod”)
Salmon	(“wild caught salmon OR “farmed salmon” OR “salmon” OR “wild-caught salmon”)
Tilapia	(“tilapia” OR “farmed tilapia” OR “wild caught tilapia” OR “wild-caught tilapia”)
Clam	(“clam” or “clams”)
Alaskan Polluck	(“Alaskan Polluck”)
Pagasius	(“pagasius” OR “basa fish” OR “catfish”)
Cephalopods	(“cephalopod” OR “cephalopods”)
Freshwater Fish	(“freshwater fish”)
Marine Fish	(“marine fish”)
Crustacean	(“crustacean”)
**Beverages:**	

Coffee	(“coffee”)
Tea	(“tea”)
Beer	(“beer”)
Wine	(“wine”)
**Processed Terms:**	
Processing	(process!) AND (food!)
Packaging/Packing	("Packaging" OR "packing") AND ((food!) OR (meat!) OR (livestock!))
Manufacturing	(Manufactur!) AND (food!)
Value-Add	((Value add!) OR (value-add!)) AND ((agri!) OR (food!))
Roast	(roast!)
Shell	(shell!)
	Ex: "Shelly", "Shelley"
Dry	(“dried” OR (dry!) OR “dries”)
	Ex: "drywall", "palm oil"
Cut	(cut!)
Dehydrate	(dehydrat!)
Homogenize	(homogeniz!)
Peel	(peel!)
Mill	(“milled” OR “milling” OR (mill!))
Grade	(grad!)
Ferment	(ferment!)
Juice	(juic!)
Can	((can!) OR "canned" OR "canning")
Chop	(chop!)

Interrater Reliability

Step	Data Source	Total Codes	Percent Agreement between Coders
1	Verite	422	99%
	IJ reports	1571	83.5%
	TIP report	45	97.8%
2	IJ reports	659	81.6%
	TIP report	108	90.7%

Weighted Average Qualitative Risk Level Calculation

Following the Social Hotspots Database (SHDB) approach, weighted average qualitative risk levels are calculated as follows:

- Known occurrences (Steps 1, 2, and 3) (85%):*
  - Very High = 4
  - High = 3
  - Medium = 2

    - Low = 1
    - Very Low = 0.5
    - NA = NA
- Governance (15%):
    - Very High = 4
    - High = 3
    - Medium = 2
    - Low = 1
    - NA = NA
- If the weighted average is >3.0 then Very High, if >2.5 then High, if >1.5 then Medium, if > 1.0 then Low, if <= 1.0 then Very Low.*

*These are the cut-points used by the Social Hotspots Database (Benoit Norris et al. 2018), with exceptions that accommodate the use of higher resolution data in our analysis. First, for Step 3 Known occurrences, each level from the GSI has been stepped down one risk tier. For example, >0.70% of people enslaved equals "Very High" in the SHDB, while it equals "High" in our analysis. Second, for Step 3 Known occurrences, we created a "Very Low" code and corresponding value (0.5) for this analysis. Finally, for the weighted average calculation, the cut-points from SHDB were adjusted to add a "Very Low" weighted average code was added. where previously "Low" applied to all values <= 1.5.

Data Quality Assessment Frameworks

Data quality assessment framework for risk coding

Score / Indicator	**1**	**2**	**3**	**4**	**5**
**Reliability of Source(s)**	Statistical study, or verified data from primary data collection from several sources or government report	Verified data from NGO report or investigative journalism publication	Qualified estimate (e.g. by expert) obtained through mini-Delphi process or external consultation	Data from non-recognized sources	Non-qualified estimate or unknown origin of source
**Temporal conformance***	Data from 2015-2019	Data from 2010-2014	Data from 2005-2009	Data from 2000-2004	Data from before 2000 or age of data unknown
**Geographical conformance****	Data from country level	Data from continent	Data from aggregated regions (e.g., HIC vs LMIC)	Global	Data from unknown geography
**Further technical conformance****	Data corresponds to same specific commodity (e.g., oranges)	Data corresponds to same generic commodity group (e.g. citrus)	Data corresponds to same sector (e.g., agriculture)	Data not specific to sector or average of multiple sectors (e.g., risk at country level)	Origin of data unclear

Data quality assessment framework for working hours

Score / Indicator	1	2	3	4	5
**Reliability of Source(s)**	Data validated with literature review.	Data not validated with literature review. Data directly from GTAP9 (2011)	Data not validated with literature review. Data from GTAP9 is an outlier (<5th or >95th percentile)	Data not validated with literature review. No country data from GTAP9.	Data not validated with literature review and origin unknown
**Temporal conformance**	Data from 2015-2019	Data from 2010-2014	Data from 2005-2009	Data from 2000-2004	Data from before 2000 or age of data unknown
**Geographical conformance**	Data from group - commodity level	Data from region-group	Data from region-sector level	Data derived from average of subsector-subregion data	Data from unknown geography
**Further Technical Conformance**	Single sector for a single commodity (i.e. Wheat)	Single sector for a group of commodities (i.e. Grains)	Single sector for multiple related commodity groups (i.e. V_F for all fruits and vegetables)	Single sector for a large number of commodity groups (i.e. Agriculture)	Commodities not classified

Data quality assessment framework for price data

Score / Indicator	1	2	3	4	5
**Reliability of Source(s)**	Verified data from primary data collection or validated using literature review	Data not validated with literature review; data not an outlier	Data not validated with literature review; data was an outlier (<5th and >95th percentile) and subject to cleaning		
**Temporal conformance**	Data from 2015-2019	Data from 2010-2014	Data from 2005-2009	Data from 2000-2004	Data from before 2000 or age of data unknown
**Geographical conformance**	Data from country level	Data from continent level	Data from aggregated regions	Data from global level	Data from unknown or distinctly different regions
**Further technical conformance**	Data from producer price	Data from export price to US or globally	Data from import price	Data from alternate source	Source of price data unclear or unknown

Combined data quality assessment framework for risk coding, working hours and price

Score / Indicator	1	2	3	4	5
**Reliability of Source(s)**	Statistical study, or verified data from primary data collection from	Verified data from NGO report or investigative	Qualified estimate (e.g. by expert) obtained through mini-Delphi process	Data from non-recognized sources	Non-qualified estimate or unknown origin of source

Risk coding data:	several sources or government report	journalism publication.	or external consultation.		
Price/working hours data:	Verified data from primary data collection or validated using literature review	Data not validated with literature review; data is not an outlier	Data not validated with literature review; data is an outlier ($<5^{th}$ and $>95^{th}$ percentile) and subject to cleaning		
**Temporal conformance**	Data from 2015-2019	Data from 2010-2014	Data from 2005-2009	Data from 2000-2004	Data from before 2000 or age of data unknown
**Geographical conformance** *Risk coding data:*	Data from country level	Data from continent level	Data from aggregated regions	Data from global level	Data from unknown geography
Working hours data:	Data from group - commodity level	Data from region-group	Data from region-sector level	Data derived from average of subsector-subregion data	Data from unknown geography
Price data:	Data from country level	Data from continent level	Data from aggregated regions	Data from global level	Data from unknown or distinctly different regions
**Further Technical Conformance** *Risk coding data:*	Data corresponds to same specific commodity (e.g., oranges)	Data corresponds to same generic commodity group (e.g. citrus)	Data corresponds to same sector (e.g., agriculture)	Data not specific to sector or average of multiple sectors (e.g., risk at country level)	Origin of data unclear
Working hours data:	Single sector for a single commodity (i.e. Wheat)	Single sector for a group of commodities (i.e. Grains)	Single sector for multiple related commodity groups (i.e. V_F for all fruits and vegetables)	Single sector for a large number of commodity groups (i.e. Agriculture)	Commodities not classified
Price data:	Data from producer price	Data from export price to US or globally	Data from import price	Data from alternate source	Source of price data unclear or unknown

References

1. World Health Organization & Center for Disease Control and Prevention. Archived: WHO Timeline - COVID-19. https://www.who.int/news/item/27-04-2020-who-timeline---covid-19 (2020).

2. Blackstone, N. T., Norris, C. B., Robbins, T., Jackson, B. & Decker Sparks, J. L. Risk of forced labour embedded in the US fruit and vegetable supply. *Nat. Food* **2**, 692–699 (2021).
