# Supplementary material for: Forced labour risk is pervasive in the US land-based food supply
Source: Nat Food. 2023 Jul 24;4(7):596–606. doi: 10.1038/s43016-023-00794-x (PMC10365991; doi:10.1038/s43016-023-00794-x)
Supplement: Supplementary file 2 — Reporting Summary [file 43016_2023_794_MOESM2_ESM.pdf]

nature portfolio

Corresponding author(s): Nicole Tichenor Blackstone

Last updated by author(s): Jun 9, 2023

Reporting Summary

Nature Portfolio wishes to improve the reproducibility of the work that we publish. This form provides structure for consistency and transparency in reporting. For further information on Nature Portfolio policies, see our Editorial Policies and the Editorial Policy Checklist.

Statistics

For all statistical analyses, confirm that the following items are present in the figure legend, table legend, main text, or Methods section.

n/a	Confirmed	
☐	☒	The exact sample size (*n*) for each experimental group/condition, given as a discrete number and unit of measurement
☒	☐	A statement on whether measurements were taken from distinct samples or whether the same sample was measured repeatedly
☒	☐	The statistical test(s) used AND whether they are one- or two-sided<br>*Only common tests should be described solely by name; describe more complex techniques in the Methods section.*
☒	☐	A description of all covariates tested
☒	☐	A description of any assumptions or corrections, such as tests of normality and adjustment for multiple comparisons
☐	☒	A full description of the statistical parameters including central tendency (e.g. means) or other basic estimates (e.g. regression coefficient) AND variation (e.g. standard deviation) or associated estimates of uncertainty (e.g. confidence intervals)
☒	☐	For null hypothesis testing, the test statistic (e.g. *F*, *t*, *r*) with confidence intervals, effect sizes, degrees of freedom and *P* value noted<br>*Give P values as exact values whenever suitable.*
☒	☐	For Bayesian analysis, information on the choice of priors and Markov chain Monte Carlo settings
☒	☐	For hierarchical and complex designs, identification of the appropriate level for tests and full reporting of outcomes
☒	☐	Estimates of effect sizes (e.g. Cohen's *d*, Pearson's *r*), indicating how they were calculated

Our web collection on statistics for biologists contains articles on many of the points above.

Software and code

Policy information about availability of computer code

Data collection	For investigative journalism data collection, Nexus Uni was used to conduct a search using a base set of labor related terms and commodity or processing terms. Raw data files were collected and managed using Microsoft Excel (v. 16.73).
Data analysis	Data processing and analysis were performed using Tableau Prep (v. 2022.3.1) and Tableau Desktop (v. 2022.2.4). TFL (Tableau Prep) files are available from the corresponding author upon reasonable request.

For manuscripts utilizing custom algorithms or software that are central to the research but not yet described in published literature, software must be made available to editors and reviewers. We strongly encourage code deposition in a community repository (e.g. GitHub). See the Nature Portfolio guidelines for submitting code & software for further information.

Data

Policy information about availability of data

All manuscripts must include a data availability statement. This statement should provide the following information, where applicable:

- Accession codes, unique identifiers, or web links for publicly available datasets
- A description of any restrictions on data availability
- For clinical datasets or third party data, please ensure that the statement adheres to our policy

Supporting data for the main figures are available as source data files. Detailed results and background data files are available for download at https://dataverse.harvard.edu/dataverse/lasting, and interactive visualizations of select results are available at https://sites.tufts.edu/lasting/data/. The supply and origin

data that support the findings of this study are available from the FAO (http://www.fao.org/faostat/en/#data, https://github.com/SWS-Methodology/faoswsAupus). The price data that support the findings of this study are available from the FAO (http://www.fao.org/faostat/en/#data) and the Global Trade Analysis Project (https://www.gtap.agecon.purdue.edu/databases/v8/). The forced labour and governance data that support the findings of this study are available from the US Department of Labor, Bureau of International Labor Affairs (https://www.dol.gov/agencies/ilab), US Department of State, Bureau of Democracy, Human Rights, and Labor and Office to Monitor and Combat Trafficking in Persons (https://www.state.gov/), Verité (https://www.verite.org/) and the Walk Free Foundation (https://www.globalslaveryindex.org/about/the-index/).

Human research participants

Policy information about studies involving human research participants and Sex and Gender in Research.

Reporting on sex and gender	NA
Population characteristics	NA
Recruitment	NA
Ethics oversight	NA

Note that full information on the approval of the study protocol must also be provided in the manuscript.

Field-specific reporting

Please select the one below that is the best fit for your research. If you are not sure, read the appropriate sections before making your selection.

☐ Life sciences ☒ Behavioural & social sciences ☐ Ecological, evolutionary & environmental sciences

For a reference copy of the document with all sections, see nature.com/documents/nr-reporting-summary-flat.pdf

Behavioural & social sciences study design

All studies must disclose on these points even when the disclosure is negative.

Study description	This study brings together existing data on commodity trade, prices, and labor intensities with newly created qualitative codes of risk to quantitatively estimate the risk of forced labor embedded in diverse food products consumed in the United States.
Research sample	Our final dataset included n=212 food products, which correspond to the U.N. Food and Agriculture Organization's Supply and Utilization Accounts (SUA) products. The dataset is considered representative for the U.S. land-based food supply, as it includes all foods in the FAO dataset with a small number of exclusions (below). Because of this complexity and the presence of products with multiple stages of processing, the total number of activity-country combinations - where “activity” stands for a supply chain stage of a food product (e.g., agriculture, first processing stage) - in the final dataset was n = 2,661.
Sampling strategy	Not applicable. Risk estimates observed in figures are provided either on a weighted basis, where the weight is proportional to the supply coming from different countries of origin, or unweighted, where the risk refers to a product from one country.
Data collection	No primary data collection with participants to report. Collection of data from investigative journalism and other publicly available sources was completed with the disclosed softwares. No experimental condition or study hypothesis to report (and thus no blinding).
Timing	Data was collected from sources between June 2021 - July 2022.
Data exclusions	Complex food products without a commodity tree structure from UN FAO were excluded due to the opacity of upstream supply chains (n = 16). All byproducts were excluded from the analysis (n= 29). Activity-country combinations were only generated for the final analysis when the importing country represented > = 1% of total supply for that activity.
Non-participation	No participants were involved in this study.
Randomization	This study was not an experiment; randomization was not applicable.

Reporting for specific materials, systems and methods

We require information from authors about some types of materials, experimental systems and methods used in many studies. Here, indicate whether each material, system or method listed is relevant to your study. If you are not sure if a list item applies to your research, read the appropriate section before selecting a response.

Materials & experimental systems

n/a	Involved in the study
☒	☐ Antibodies
☒	☐ Eukaryotic cell lines
☒	☐ Palaeontology and archaeology
☒	☐ Animals and other organisms
☒	☐ Clinical data
☒	☐ Dual use research of concern

Methods

n/a	Involved in the study
☒	☐ ChIP-seq
☒	☐ Flow cytometry
☒	☐ MRI-based neuroimaging
